# The Role of Motivation and Anxiety on Error Awareness in Younger and Older Adults

**DOI:** 10.3389/fpsyt.2021.567718

**Published:** 2021-02-19

**Authors:** Elisa Di Rosa, Fabio Masina, Antonino Vallesi, Daniela Mapelli

**Affiliations:** ^1^Department of General Psychology, University of Padua, Padua, Italy; ^2^School of Psychology, Keele University, Newcastle-under-Lyme, United Kingdom; ^3^Istituto di Ricovero e Cura a Carattere Scientifico, San Camillo Hospital, Venice, Italy; ^4^Department of Neuroscience & Padova Neuroscience Center, University of Padova, Padova, Italy; ^5^Brain Imaging and Neural Dynamics Research Group, Istituto di Ricovero e Cura a Carattere Scientifico, San Camillo Hospital, Venice, Italy

**Keywords:** motivation, anxiety, error awareness, aging, reward

## Abstract

Aging is associated with several changes in cognitive functions, as well as in motivational and affective processes, which in turn interact with cognitive functions. The present study aimed to investigate error awareness (EA), which declines with aging, in relation to motivation and anxiety. Adopting an experimental task, we firstly tested the hypothesis that EA could be enhanced through reward motivation. Secondly, we explored the relation between state and trait anxiety and EA, investigating the hypothesis of an association between EA and anxiety, and between anxiety and the potential benefit of motivation on EA. Thirty healthy younger (age range: 19–35 years; mean age 25.4 ± 5.1; 10 M) and 30 healthy older adults (age range: 61–83 years; mean age 69.7 ± 5.5; 12 M) took part in the study and performed both the classic Error Awareness Task (EAT) and one experimental task, called the Motivational EAT. In this new task, motivational incentives were delivered after aware correct responses and aware errors. For every participant, standard measures of state and trait anxiety and cognitive functions were collected. Confirming the presence of a significant age-related EA decline, results did not reveal any influence of reward motivation on EA, nor any relation between EA and anxiety. However, both younger and older adults had longer response times (RTs) and made more errors during the Motivational EAT, with the more anxious participants showing the greater RT slowing. Findings suggest that reward motivation might not be always beneficial for cognitive performance, as well as that anxiety does not relate to EA capacity. Results also recommend further investigation, as well as the assessment of EA in patients with either motivational deficits like apathy, and/or with anxiety disorders.

## Introduction

A growing body of evidence suggests that performance monitoring and error awareness (EA) are negatively impacted by the aging process (1–9). As Harty et al. (1) highlighted, “this phenomenon is particularly concerning in light of the associations between impaired awareness of cognitive functioning and engagement in risky behavior, increased care-giver burden, poor motivation for treatment and poor general prognosis,” and would therefore benefit from further investigation.

While the neural underpinning of this phenomenon has been the focus of few recent studies (1, 5, 7, 8), only one work (2) explored the possibility to counteract age-related EA decline. In detail, Harty et al. (2) suggested that anodal transcranial direct current stimulation (tDCS) over the right lateral prefrontal cortex brings to an improvement of EA in older adults. However, sometimes tDCS can be difficult to employ with older adults, because they may not fulfill all the inclusion criteria for the use of non-invasive brain stimulation [see (10)].

According to the Value-Based Cognitive Control framework (11–13), the presence of motivational incentives, like rewards, has the capacity to increase the motivational value of cognitive control, and to consequently bring to a cognitive performance enhancement. Despite the precise neural mechanism behind motivation-cognition interaction is still not clear, is now well established that dopamine plays a key in performance enhancement [(13); see also (14)], either by its tonic release in the prefrontal cortex (PFC), which might facilitate cognitive stability, or by its phasic release in the striatum, which may facilitate cognitive flexibility (13).

Beneficial effects of reward motivation on cognitive performance have been reported in both younger and older adults [for a recent review, see (15); see also (16–21)], with relevant advancement in aging research, increasingly aimed to understand the mechanisms behind age-related cognitive decline, and consequently to find effective strategies to counteract it.

However, to the best of our knowledge, nobody has yet investigated the effect of reward motivation on EA and, more importantly, in reducing the EA age-related decline.

Hence the first aim of the present study was to test the hypothesis of a positive effect of reward motivation on EA and, more specifically, on the age-related EA decline. To test this hypothesis, we designed an experimental EA task, the Motivational Error Awareness Task (EAT), introducing performance contingent feedback and positive motivational incentives (virtual monetary reward), and we tested both younger and older adults. By comparing their performance at this new task with the one at the Classical EAT [reported in our previous work, see (4)] we predicted to find a significant EA enhancement, as well as a reduction of the age-related EA decline, in the Motivational EAT.

The present work was also guided by a second aim, which was the investigation of the role of anxiety on EA and on the age-related EA decline. This second aim was inspired by several sources of evidence and theoretical frameworks indicating the existence of a relation between anxiety, cognitive performance, and aging.

For what concerns the relation between anxiety and cognitive performance, as recently summarized by Hoshino and Tanno (22), several studies demonstrate that trait anxiety can influence various cognitive processes, from early perceptual detection stages to higher-order processes, such as cognitive control. More specifically, according to both the Attentional Control Theory (ACT) by Eysenk et al. [(23); see also (24)] and the Dual Mechanisms of Control framework (DMC) (25, 26), elevated levels of trait anxiety decrease the functional efficiency of executive control, and more specifically of the proactive control mode (25, 26). Cognitive control is actually achieved through two distinct modes: proactive, which involves active maintenance of rules and goals, and reactive, which involves allocating attention to rules and goals on an as-needed basis, once a problem (such as the occurrence of a conflict, or an error) has arisen (25).

According to Braver (26), while non-anxious individuals are able to alternate flexibly between reactive and proactive control modes in accordance with changing task demands, the distraction caused by worries would make anxious individuals less efficient in implementing proactive control, and therefore more dependent on a compensatory increase of reactive control, especially when salient events, such as errors, occur [see also the *Compensatory Error Monitoring Hypothesis* by (27)].

Based on this first set of evidence and theoretical frameworks, we could therefore predict that higher levels of trait anxiety might be associated with higher error rates, as result of decreased levels of proactive control, but also with higher levels of EA, as result of a compensatory enhancement of reactive control. This prediction however, to the best of our knowledge, has not yet found a demonstration. Actually, to the best of our knowledge, so far only one study has explored the relation between anxiety and EA, without finding any significant association between the two (3). Harty et al. (3), however, employed the Hospital Anxiety and Depression Scale (HADS) (28), which asks participants to evaluate how they felt in the past week, and therefore does not assess trait anxiety.

Hence, we decided to further test the hypothesis of a positive association between trait anxiety and EA employing a different measure, such as the State-Trait Anxiety Inventory—STAI (29).

Furthermore, based on the literature suggesting a possible relation between anxiety and the age-related decline in cognitive performance (30–32) we also wanted to investigate if anxiety was in some way associated with the age-related decline on EA. In this case, given the fact that the direction and the temporal dynamics of the relation between the age-related cognitive decline and anxiety are not clear yet, we did not have a specific prediction.

Finally, as a third exploratory aim, we also wanted to investigate if anxiety would be related to the potential effect that the motivational manipulation employed in the present study might have had on EA. Actually, some recent studies suggest that motivation is an important variable in explaining the relation between trait anxiety and cognitive performance, because high trait-anxious individuals would be more apprehensive about their performance (33), and therefore more motivated to invest further cognitive effort when performing a task (22, 34). Our last prediction was therefore to find a positive association between trait anxiety and the potential beneficial effect of motivation on EA.

## Methods

### Participants

Sixty healthy participants were recruited^1^: 30 younger adults (age range: 19–35 years; mean age 25.4 ± 5.1; 10 M) and 30 older adults (age range: 61–83 years; mean age 69.7 ± 5.5; 12 M). Inclusion criteria were: an age between 18–35 (younger adults) and 60–85 (older adults) years; the availability to take part in a two-session experiment; a normal or corrected-to-normal vision; the ability to sign the informed consent. Exclusion criteria were: present or past neurological or psychiatric diseases; use of neurological or psychiatric medications; a score at the Montreal Cognitive Assessment (MoCA) (35) under the Italian cut-off [i.e., 15.5 (36, 37)] (see Table 1). Participants received no compensation for taking part in the study. Written informed consent was obtained from all participants. The study was conducted in accordance with the Helsinki Declaration on human rights and was approved by the Ethics Committee of the School of Psychology at the University of Padua.

**Table 1:** Mean scores obtained at the standard psychological and cognitive tests, and years of education, of both groups. Standard deviations are in parenthesis.

	**Younger adults**	**Older adults**
MoCA	28.1 (1.6)	25.9 (2.5)
STAI-S (Classical EAT)	34.2 (7.9)	32.6 (6.2)
STAI-S (Motivational EAT)	32.2 (4.8)	32.3 (6.0)
STAI-T	40.7 (9.7)	36.0 (8.7)
TIB	106.8 (4.7)	111.2 (8.6)
CRIq	92.7 (6.3)	105.3 (25.3)
Short term memory (mean score)	16.2 (4.2)	10.8 (2.5)
TMT B-A	39.0 (15.9)	63.1 (42.7)
Years of education	15.1 (2.7)	11.3 (5.5)

### Experimental Task Procedure

To test the hypothesis of a positive effect of reward motivation in EA, the performance at two different versions of the Error Awareness Task (EAT) (38) was compared.

**Figure 1:**
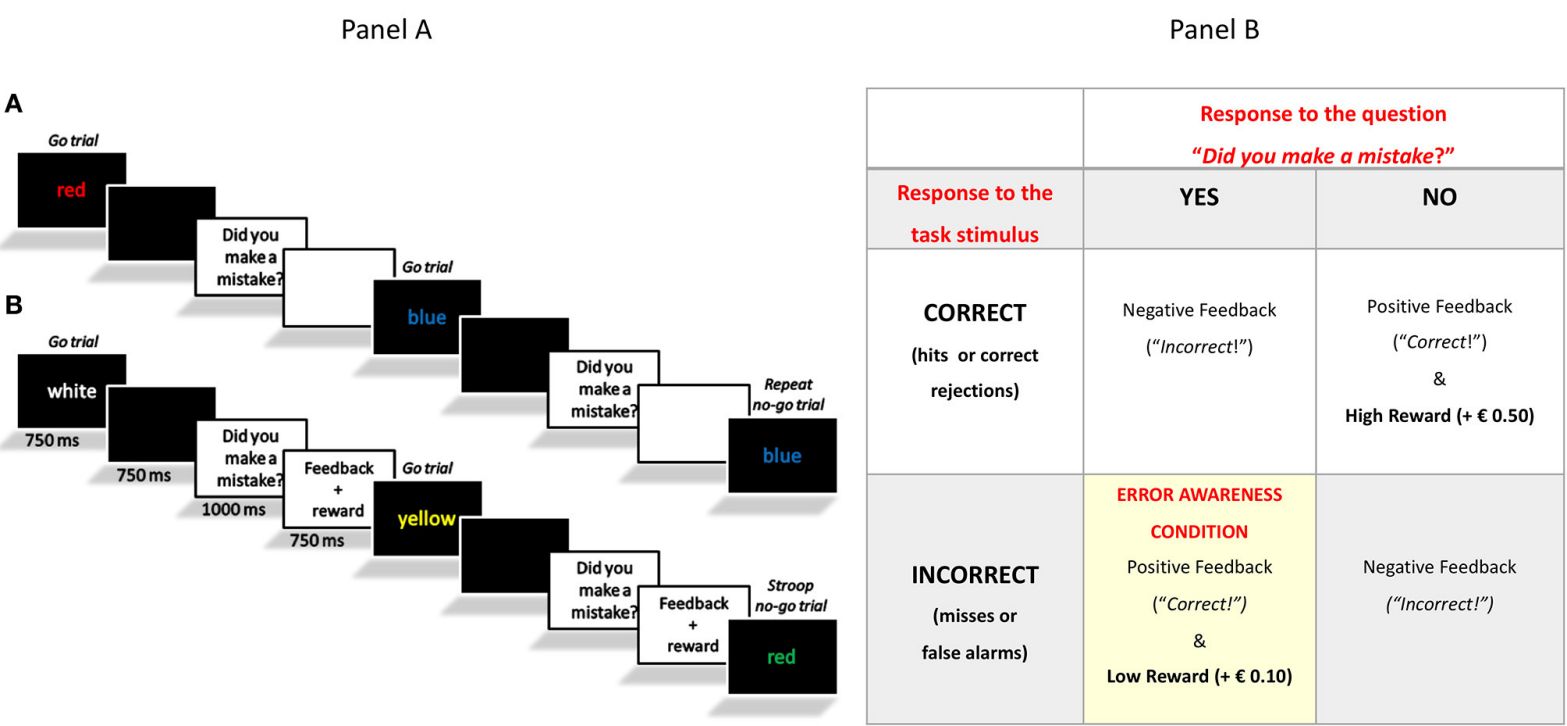
**(Panel a)** Schematic representation of the experimental tasks. In Sessions 1 and 2, the Classical EAT **(A)** and Motivational EAT **(B)** were counterbalanced across participants. Only in the Motivational EAT participants received a feedback after a response and a reward after a correct response or a signaled error. **(Panel b)** Schematic representation of the rewarding scheme adopted in the Motivational EAT.

In one version of the task, which will be hereby called the “Classical EAT” (4), a serial stream of single color words was presented at the center of the screen. Participants were asked to respond with a single-speeded press (“3” on the keyboard) when the word and its color font were congruent (go trials). In addition, they were trained to withhold the response when the word and its color font were incongruent (Stroop no-go trials), or when the word was presented twice in a row (repeat no-go trials). Following the offset of the word, the sentence “Hai commesso un errore?” [in English: “Did you make a mistake?”] prompted participants to monitor their performance online. In case participants realized they had made a mistake, they were required to press an error button (space bar), in order to signal it (see Figure 1A). The data concerning the Classical EAT performance of the overlapping participants have been reported in our previous study (4).

The second version of the task was designed for this specific study and was called the “Motivation EAT.” It was identical to the Classical EAT, except for the presence of feedbacks and virtual rewards. Specifically, we decided to use both performance-dependent (positive or negative) feedback and virtual monetary incentives (high and low reward) in order to motivate our participants to perform at their best and, moreover, to motivate them to be aware of their own performance, and therefore their errors. For this reason, we did not directly reward/give a feedback after a stimulus response per se, but we delivered reward and feedback only after the response to the question “Did you make a mistake?” (see Figure 1A).

More in detail, in case of correct responses to stimuli and correct responses to the question “did you make a mistake,” the feedback “Corretto!” [“Correct!”] was presented, as well as a virtual reward of € 0.50. On the contrary, in case participants did not respond in the correct way to the question “did you make a mistake?,” the feedback “Sbagliato!” [“Incorrect!”] appeared, but no losses were applied. If participants made a mistake (wrong response to the stimulus) and responded “yes” to the question “did you make a mistake?,” showing therefore error awareness, they received a virtual reward of € 0.10. Four conditions where therefore possible, as summarized in Figure 1B.

After receiving a reward, the information about the updated total wins appeared at the bottom of the screen. At the end of the experiment, each participant received information about the total wins.

The purpose of associating correct task responses with higher reward, and EA with lower reward, was to motivate participants to enhance performance monitoring without increasing error rate. At the same time, the choice to use only positive incentives and to give only negative feedback (and not negative incentives/punishment) after incorrect responses, was made because of the older adults' selective sensitivity to gains, and reduced sensitivity to losses [see (39)].

In both versions of the task, 675 stimuli were presented, in three blocks of 225 trials (200 go trials and 25 no-go trials, of which 12 Stroop no-go trials and 13 repeat no-go trials; see Figure 1A). The tasks were administered in two separate sessions and in a counterbalanced order.

The experiments were run by E-Prime software (version 2.0 Psychology Software Tools, Pittsburgh, PA) installed on a personal computer equipped with a 15″ monitor.

Dependent variables considered as indices of performance were the correct response times (RTs) >100 ms, the accuracy rate at both go and no-go stimuli, and EA, calculated as the percentage of correctly signaled commission errors on the total number of commission errors (40).

### Psychological Assessment

Both younger and older participants were asked to take part in a standard “paper and pencil” testing phase, where state and trait anxiety were collected. Specifically, at the end of each of the two sessions, participants completed the State-Trait Anxiety Inventory—State (29), while at the end of the second experimental session only, they also completed the State-Trait Anxiety Inventory—Trait (29).

Furthermore, we also employed the following standard cognitive tests: Verbal Short-Term Memory Test [immediate and delayed recall; both from ENB 2 (41)], in order to assess short-term memory; Trail Making Test A and B [from ENB 2 (41)], in order to assess general speed and task switching; Test di Intelligenza Breve—TIB [(42)—Italian equivalent of the National Adult Reading Test (43)], in order to estimate IQ; Cognitive Reserve Index questionnaire (CRIq) (44), in order to estimate cognitive reserve.

### Data Analysis

One participant in the older group was excluded from analyses because of technical difficulties during the task, leading to a total sample of 59. The normality of the distribution of each variable of interest was assessed using Kolmogorov-Smirnoff test. Results indicated that both EA and the scores at the standardized psychological and cognitive tests were not normally distributed, while RTs and Accuracy rates resulted to be normally distributed in both tasks (minimum *p* > 0.20 at the Kolmogorov-Smirnoff test).

Therefore, within-group differences in the EA, measured in the two experimental conditions (Classical EAT vs. Motivational EAT) were assessed using Wilcoxon signed-rank test, while between group differences in terms of EA, as well as in terms of state and trait anxiety and cognitive functions were assessed using Mann–Whitney *U*-test. Based on Bonferroni correction for multiple comparisons, the significant *p*-value for these non-parametric tests was set equal to 0.004 (rounding down 0.05/12).

Between and within group differences in terms of RTs and accuracy were assessed by conducting two mixed ANOVAs, considering as within-subjects factor the *Task* (Classical EAT vs. Motivational EAT) and as between-subjects factor the *Group* (younger vs. older adults). Partial eta squared (η_*p*_^2^) was used as measure of effect size. Bonferroni correction was employed in case *post-hoc* comparisons were performed.

Correlations between EA, measured during the Classical EAT, and scores obtained at the standardized anxiety and cognitive tests, including the MoCA, were assessed using two-tailed Spearman's rank correlation coefficient. Information about age and education was also included in the correlation analysis. Correlations were conducted considering the total sample (*N* = 59). However, of the older adults group, three participants did not complete the STAI state scale, while one participant did not complete the TMT. Hence, in the analyses that considered these two tasks, *N* was respectively equal to 56 and 58. Based on Bonferroni correction, significant *p*-value for the correlations was set equal to 0.005 (0.05/10).

## Results

Table 1 reports the scores obtained at the standard psychological and cognitive tasks, together with information about education. EA, mean correct RTs and accuracy rates, as a function of task and group, are reported in Table 2A.

**Table 2A:** Mean correct RTs (milliseconds), accuracy rates (%), and EA (%), as a function of task and group. Standard deviations are in parenthesis.

**Group**	**Classical EAT**			**Motivational EAT**		
	**RTs**	**Accuracy**	**EA**	**RTs**	**Accuracy**	**EA**
Younger adults	483.5 (59.7)	94.5 (2.3)	88.5 (7.7)	499.5 (76.7)	94.1 (2.8)	80.7 (13.9)
Older adults	630.4 (91.9)	95.5 (2.0)	57.6 (2.1)	654.26 (90.6)	94.8 (1.8)	56.7 (2.4)

Results confirmed the presence of lower EA levels in older adults, when compared with the younger ones, in both tasks (Classical EAT: *U* = 63.5, *p* < 0.0001; Motivational EAT: *U* = 160, *p* < 0.0001). No significant results emerged in terms of EA when assessing the differences between the two tasks in both groups.

Older adults, when compared with the younger ones, had a significantly lower performance at the MoCA test (*U* = 194.5, *p* < 0.0001) and at the Short-term memory^2^ test (*U* = 130.5, *p* < 0.0001). No significant differences were revealed between younger and older adults in terms of state and trait anxiety and in the other cognitive tests employed (TMT B-A, TIB, and CRIq).

Results of the ANOVA on RTs confirmed the age-related decline in response speed, with significantly longer RTs in older adults, when compared with the younger ones, independently of the task [*F*_(1,57)_ = 57.21; *p* < 0.0001; $\eta_{p}^{2}$ = 0.98]. Moreover, results also showed a significant difference when comparing the two tasks, with longer RTs during the Motivational EAT with respect to the Classical EAT, independently of the group [*F*_(1,57)_ = 9.23; *p* < 0.005; $\eta_{p}^{2}$ = 0.13]. Finally, a significant difference between the two tasks also emerged in terms of accuracy [*F*_(1,57)_ = 5.19; *p* < 0.05; $\eta_{p}^{2}$ = 0.08], with lower accuracy rates at the Motivational EAT, if compared with the Classical EAT, independently of the group.

Results of the correlation analysis (Table 2B) revealed that EA, assessed with the Classical EAT, was significantly related with both age (Rho = −0.671; *p* < 0.0001) and MoCA scores (Rho = 0.472*; p* < 0.0001). A positive significant association was also revealed between EA and short-term memory test score (Rho = 0.434; *p* < 0.005), while a negative significant association was present between EA and TMT B-A score (Rho = −0.411; *p* < 0.005).

**Table 2B:** Correlations between EA (Classical EAT), age, education and the scores at the standard cognitive and psychological tests.

	**Age**	**Education**	**MoCA**	**STAI-S**	**STAI-T**	**TIB**	**CRIq**	**Short term memory**	**TMT B-A**
EA	Rho = −0.67^*^ p < 0.001	Rho = 0.36 *p* = 0.006	Rho = 0.47^*^ *p* < 0.001	Rho = −0.02 *p* = 0.89	Rho = 0.1 *p* = 0.47	Rho = −0.13 *p* = 0.33	Rho = −0.04 *p* = 0.77	Rho = 0.43^*^ *p* = 0.001	Rho = −0.41^*^ *p* = 0.001

* *refers to a p < 0.005*.

No significant correlations emerged between EA and either state or trait anxiety (see Table 2B).

Based on the results obtained when comparing the Classical EAT and the Motivational EAT, namely the increase of both RTs and error rates, we decided to perform an additional a posteriori correlation analysis, to investigate possible associations between the RT and accuracy between-task differences, on the one side, and the scores obtained at the standard tests, on the other side.

**Figure 2:**
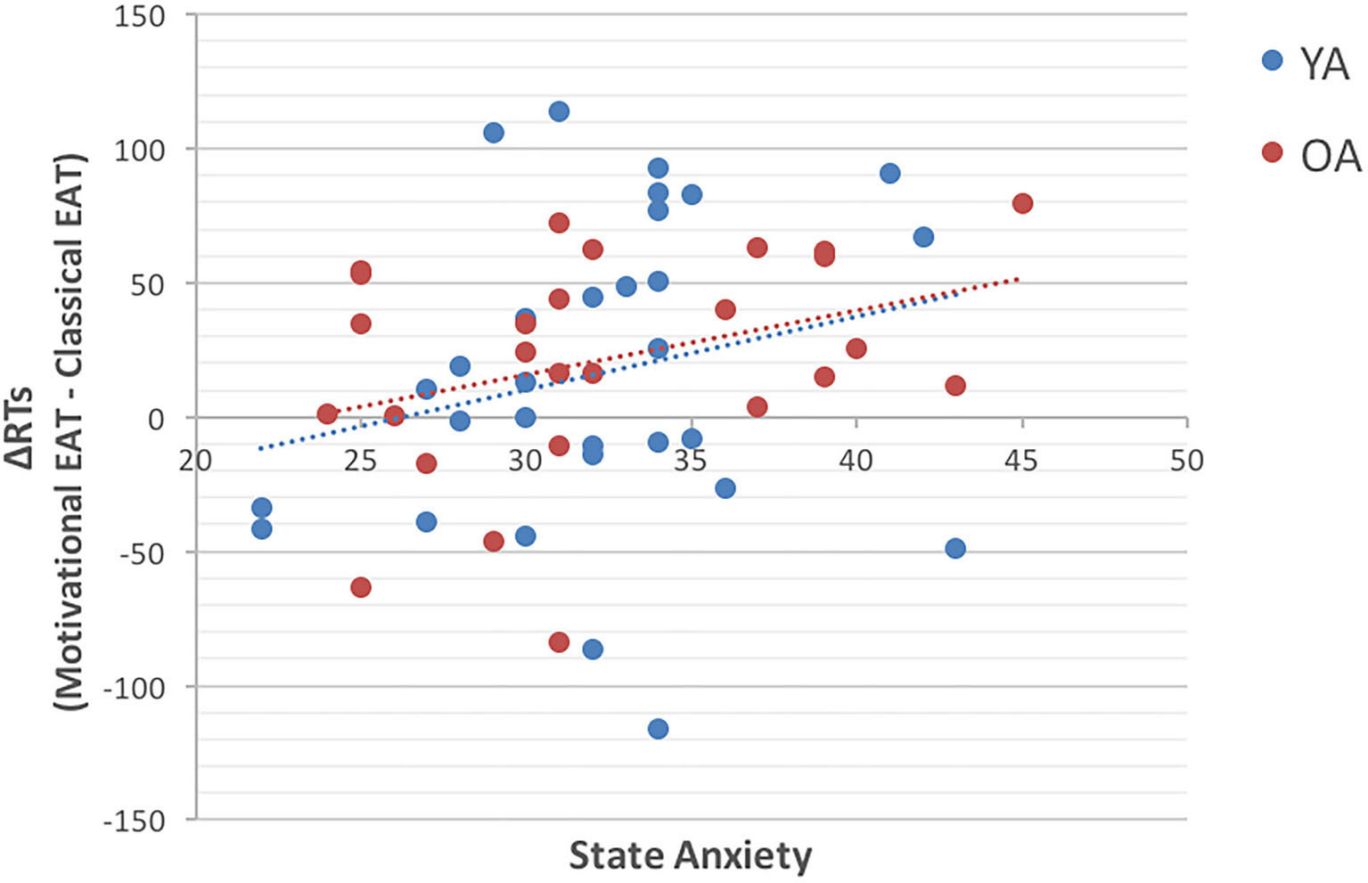
The scatterplot represents the correlation between State Anxiety (on the X axis) and the ΔRT (on the Y axis) obtained by comparing the two experimental tasks. Precisely, ΔRT = (RTs in Motivational EAT – RTs in the Classical EAT). The blue dots represent younger adults (YA), while red dots represent older adults (OA). Results suggest that participants with higher state anxiety are the ones who show longer RTs in presence of reward.

Results showed that only one correlation met conventional statistical significance levels (*p* < 0.05), and precisely the one between state anxiety, measured in the Motivational EAT session, and the RT difference between the two tasks (Rho = 0.30; *p* < 0.05), with greater slowing in participants with higher state anxiety levels (Figure 2).

Because this result would not survive after applying multiple comparisons correction, we will consider and discuss this last result only for hypothesis generation for follow-up studies.

## Discussion

The first aim of the present study was to test the hypothesis of a positive effect of reward motivation on EA and, moreover, on age-related EA decline. Our prediction was that the presence of motivational incentives would have improved the EA levels and reduced the age-related EA decline.

As a second aim, the present study also explored possible associations between anxiety, especially trait, and EA. Our prediction was to find a positive association between the two. As a third exploratory aim, we investigate the possible interaction between anxiety, motivation and EA.

Results will be therefore discussed according to these three aims.

### Reward motivation and EA

Differently from our prediction, EA did not improve when motivational incentives were associated to correctly detected errors, neither in younger nor in older adults. Moreover, the association of higher incentives with correct responses did not improve accuracy either, but on the contrary had a negative effect on it and on RTs, with both younger and older adults showing a higher error rate and slower responses during the Motivational EAT.

A first possible explanation for the lack of a reward effect is represented by the low difficulty of our tasks. First of all, the high accuracy rates that both younger and older adults showed on the Classical EAT indicate that our baseline experimental paradigm might have been not challenging enough for the purposes of the present study. Introducing motivational incentives in a more challenging task may have elucidated further effects of reward on accuracy.

Another possible explanation is that reward stimuli, being presented in the inter-trial intervals, distracted participants. This hypothesis would be in line with a series of previous works, which suggested that reward signals can automatically influence visual attention beyond, and sometimes against, the strategic control of goal-directed attention (45–47). This phenomenon is also explained in the well-established “distraction theory” (48, 49), according to which the presence of rewards could represent a distracting environment and may draw the performers' attention away from skill execution, causing the “choking under pressure” phenomenon (50).

Finally, the reward manipulation could have not be effective in enhancing task performance because of an inappropriate reward delivery timing. Actually, it has been shown that the effects of reward on cognitive performance also depend on when the information about the reward is presented. Specifically, while a pre-stimulus reward-cue seems to have positive effects on cognitive performance, a reward presented together with the stimulus can have detrimental effects on visual attention (51).

### Anxiety and EA

Results of the present study did not reveal the presence of any correlation between state or trait anxiety and EA. On the contrary, significant correlations emerged only between EA and age, and between EA and scores obtained at the standard cognitive tests. Specifically, we report a significant positive association between EA and both the MoCA and short-term memory test performance, such as individuals with better general cognitive performance and with a more efficient short-term memory, are also more aware of their mistakes. We also found a negative association between EA levels and the task switching capacity, estimated through the TMT B-A. This further confirm the association with EA and high order cognitive abilities. We did not find any association between anxiety (state and trait) and any of the scores obtained at the other cognitive tests employed.

Furthermore, while we found a significant difference in terms of EA between younger and older adults, coherently with the literature (1–3, 5–9) and we found predictable age-group differences in general cognitive performance (i.e., MoCA) and short-term memory, we did not find any significant difference between the two groups in terms of state or trait anxiety.

Therefore, this second set of results suggests that state or trait anxiety might not have a role in modulating EA, and that age-related EA decline should be considered as a consequence of a more general age-related cognitive decline, without any association with state or trait anxiety.

### Possible Interaction Between Motivation, Anxiety and Cognitive Performance

Results of the exploratory analysis, conducted in order to better understand the unpredicted higher RTs and error rates during the Motivational EAT, indicated that individuals who showed the longer RTs during the Motivational EAT were also the ones with higher level of state anxiety.

This result, although it has to be considered with caution, would be in line with the above-mentioned Compensatory Error Monitoring hypothesis (27), which suggests that anxious individuals need to make a greater effort in order to maintain task-related goals and a good level of performance. Interestingly, this theory also suggests that this greater amount of effort would be necessary to compensate for the distracting effect of worry, and would translate in a reactive control mode, which is more time consuming and could therefore explain the longer RTs.

We could therefore speculate that if our reward manipulation actually acted also as a distractor for our participants, this was particularly true for the ones with higher state anxiety.

At the same time, the possibility to obtain a reward could have been itself a reason to worry, causing therefore greater slowing in individuals who tend to be more anxious.

### Limitations and Future Directions

Some limitations should be noted when considering our results.

First of all, the experimental task designed for the present study did not allow us to clearly disentangle the effect of reward stimuli on motivation from the one that it probably had on attention resources. As previously explained, the post-response reward presentation could have actually distracted our participants, leading to longer RTs and higher error rates. To overcome this limitation, future study might want to test a different timing of the motivational manipulation, presenting for example a pre-stimulus reward-cue, or directly employing a block design, comparing counterbalanced reward and non-reward task blocks.

Secondly, as previously mentioned, both our tasks might have been not challenging enough for the purposes of the present study. A more difficult task, or a task tailored on an individual baseline performance, may have elucidated further effects and could represent a future effort in order to further investigate if motivation might have a potential beneficial effect on EA. To this aim, the introduction of a standard test of reward sensitivity, like the BIS/BAS scale (52), as well as the recruitment of a larger sample, would be helpful as well.

An interesting and extremely valuable future direction, in our opinion, would be also represented by the study of EA in clinical samples, such as patients with either motivational deficits like apathy, and/or with anxiety disorders. Assessing EA both alone and in relation to the presence of motivational incentives in these populations could actually represent an ideal condition, which would allow to better understand the interaction between EA, motivation and anxiety, with great benefit from both a theoretical and a clinical point of view.

## Conclusions

To the best of our knowledge, this work represents the first study investigating the effect of reward motivation on EA as well as the relation between EA and trait anxiety, in both younger and older adults. Taken together, results of the present study confirm the presence of an age-related EA decline and suggests its strict relation with the general cognitive status as well as with the short-term memory capacity. Results also show the absence of a significant relation between state and trait anxiety and EA, as well as the lack of effect of reward motivation on EA.

We therefore hope that this study will inspire many others, which, by overcoming the above-mentioned limitations, should be aimed to add new evidence in this research field, in order to clearly establish if and how EA can be enhanced through reward motivation. Moreover, we hope that this study will be considered also for its practical implications, such as the need to find effective strategies to enhance EA as well as the importance of assessing EA in the clinical practice. In our opinion, because a deficient EA would have detrimental effects on any rehabilitation outcomes, EA assessment should be present together with both cognitive and psychological tests in every clinical assessment, especially if the patient is an older adult, and particularly before any rehabilitation and treatment procedure begins. The introduction of an EA assessment in the clinical practice would in this way improve the effectiveness of any interventional approach, and therefore represent an important development in psychiatry, as well in clinical psychology and neuropsychology.

## Data Availability Statement

The raw data supporting the conclusions of this article will be made available by the authors, without undue reservation.

## Ethics Statement

The studies involving human participants were reviewed and approved by Ethics Committee of the School of Psychology at the University of Padua. The patients/participants provided their written informed consent to participate in this study.

## Author Contributions

ED, FM, and DM: study's conceptualization. ED and FM: data collection and analysis. ED, FM, AV, and DM: data interpretation, writing up, and revision of the manuscript. All authors have approved the final manuscript.

## Conflict of Interest

The authors declare that the research was conducted in the absence of any commercial or financial relationships that could be construed as a potential conflict of interest.

## Footnotes

^1^Participants in the present study were the same as in Masina et al. (4) with the exception of two elderly participants.

^2^Average between immediate and delayed recall scores.
